# Potential biomarkers that discriminate rheumatoid arthritis and osteoarthritis based on the analysis and validation of datasets

**DOI:** 10.1186/s12891-022-05277-x

**Published:** 2022-04-04

**Authors:** Le Kang, Chengqian Dai, Lihong Wang, Xinling Pan

**Affiliations:** 1 Department of Biomedical Sciences Laboratory, Affiliated Dongyang Hospital of Wenzhou Medical University, Dongyang, Zhejiang China; 2 Department of Orthopedics, Affiliated Dongyang Hospital of Wenzhou Medical University, Dongyang, Zhejiang China

**Keywords:** Rheumatoid arthritis, Osteoarthritis, Differentially expressed genes

## Abstract

**Background:**

Rheumatoid arthritis (RA) and osteoarthritis (OA) share some similar arthritic symptoms, but different mechanisms underlie the pathogenesis of these two diseases. Analysis of differentially expressed molecules in rheumatoid arthritis and osteoarthritis may assist in improving diagnosis and treatment strategies in clinical practice.

**Methods:**

Microarray and RNA-seq data were acquired from the gene expression omnibus database. Differentially expressed genes (DEGs) were identified using Bioconductor packages. Receiver operating characteristic curves were plotted to assess performance. Gene ontology enrichment analysis was conducted using the clusterProfiler application. During validation, synovial fluid was harvested from patients who had undergone in-hospital joint replacement, in which the expression of proteins was measured using enzyme-linked immunosorbent assays.

**Results:**

Compared with OA samples, RA samples showed 14 genes to be upregulated and 3 to be downregulated. Gene ontology analysis indicated that DEGs principally included molecules responsible for the regulation of a synovial tissue inflammatory response. Seven genes displayed a good discriminatory power with an AUC higher than 0.90. *ADAMDEC1* was the biomarker that most clearly discriminated RA from OA in the database, exhibiting an AUC of 0.999, a sensitivity of 100%, and a specificity of 97.8%. Following validation, the expression levels of ADAMDEC1 in the synovial fluid from RA patients were significantly higher than those in the synovial fluid from OA patients (*P* < 0.05). At the cut-off value of 1957 pg/mL, ADAMDEC1 expression in the synovial fluid discriminated RA from OA with an AUC of 0.951, a specificity of 88.6%, and a sensitivity of 92.9%.

**Conclusion:**

The differential expression of genes in RA compared with OA indicates potential targets for molecular diagnosis and treatment. The presence of ADAMDEC1 in synovial fluid is a good biomarker of RA.

**Supplementary Information:**

The online version contains supplementary material available at 10.1186/s12891-022-05277-x.

## Introduction

Rheumatoid arthritis (RA) is a common systemic autoimmune disease. It is characterized by chronic synovial inflammation and hyperplasia in addition to joint destruction, ultimately leading to disability. RA currently affects 0.1%–0.5% of adults [1]. It is approximately three times more common in women than in men, increasing in severity with age, but with a higher incidence in women aged more than 65 years [1, 2]. RA joints are characterized by inflammation of the synovium, which leads to destruction of the articular cartilage and underlying bone when the disease progresses without efficient control. Synovial hyperplasia results from synovial outgrowth or villi which consist of macrophages, cells of the synovial lining, lymphocytes, and blood vessels [3].

Osteoarthritis (OA) is a common form of chronic arthritis in the aged population [4]. There are multiple risk factors for the development of OA, including aging, obesity, and genetic factors [5]. OA is a degenerative joint condition, primarily affecting the hands, hips, and knees in which there is a loss of articular cartilage, synovial membrane dysfunction, sclerosis of the subchondral bone, and osteophyte formation combined with depletion of matrix proteins driven by proteases [6, 7].

Although mechanisms of pathogenesis of RA and OA are different, they share similar symptoms if no treatment is administered, involving principally joint dysfunction and soreness. The diagnosis and assessment of RA and OA mainly use semi-quantitative methods of diagnosis, including radiological imaging, patient symptom determination, joint damage assessment, and physical function assessment [6], which are limited in patients without classical changes (at initial stage). Although an increasing use of anti-rheumatic agents has been applied for controlling progression of RA compared with only symptomatic intervention for patients with OA, replacement of joints would be adopted in both disease groups when the involved joints lose function.

Therefore, the present study aimed to confirm the differentially expressed genes (DEGs) between RA and OA using bioinformatics analysis. An understanding of DEGs may improve diagnosis and treatment strategies available for patients in clinical practice, and may also lead to the elucidation of the different mechanisms underlying the pathogenesis of RA and OA.

## Materials and methods

### Data resource

The National Center for Biotechnology Information's Gene Expression Omnibus (GEO) database was used to retrieve appropriate microarray datasets, including the following accession numbers: GSE55235, GSE55457, GSE55584, GSE12021, GSE1919, and GSE36700. All microarray data were from the synovial tissue of human knee joints. Additional information about this resource is available from https://www.ncbi.nlm.nih.gov/geo/[8–12].

The gene transcription profile of GSE89408 deposited by Walsh et al*.* that was downloaded from the GEO repository was generated using a GPL11154 platform (Illumina HiSeq 2000). The gene transcription dataset satisfied the inclusion criteria (synovial tissue from OA and RA patients) and was therefore included in the present study [13].

### Data reprocessing and identification of DEGs

The raw microarray datasets and high-throughput sequencing data files were acquired from different platforms. Hence, the processed data within the GEO datasets were downloaded and used for subsequent analyses. Bioconductor applications within the R language environment (version 4.0.2) were used to perform data analysis. Microarray datasets with raw data (.CEL files) were corrected for background with a robust multi-array analysis (RMA) algorithm and quantile normalized in the R software [14]. High-throughput sequencing data normalization was conducted using the Affy package for Affymetrix oligonucleotide arrays, Empirical Analysis of Digital Gene Expression Data in R (edgeR), and differential gene expression analysis using the negative binomial distribution (DESeq2) package in R software.

Linear Models for Microarray Data (limma), edgeR, and DESeq2 packages were run in the Bioconductor environment to confirm DEGs by comparing expression values in synovial RA tissue with those in the OA tissue. Corresponding P-values of gene expression were defined after t-tests were performed. An adjusted P-value < 0.05 and log2 fold change (FC) > 1 was selected as cut‐off criteria. Finally, the DEGs in every dataset were analyzed, from which the intersection of overlapping expression of the DEGs across the seven datasets was identified [15].

### Gene ontology (GO) enrichment analyses of DEGs

Annotations of the cellular components (CCs), biological processes (BPs), and molecular functions (MFs) of the DEGs were analyzed using cluster Profiler package in Bioconductor to perform GO enrichment analysis (https://git.bioconductor.org/packages/clusterProfiler). 

### Receiver Operating Characteristic (ROC) analysis

ROC curves were plotted using the “pROC” package in R software [16]. Optimal sensitivity and specificity were calculated depending on the different cut-off values of gene expression, allowing the discrimination between RA and OA. ROC curves were interpreted in terms of sensitivity, specificity, and area under the ROC curve (AUC).

### Synovial fluid collection and measurement of ADAMDEC1 protein

Synovial fluid samples were collected from patients who had undergone joint replacement surgery from May 2016 to December 2019. The diagnosis of OA and RA was based on laboratory examinations, radiological examinations, and described symptoms.

The protein (ADAMDEC1) encoded by the DEG with the most discrimination power was validated in the synovial fluid. After dilution, the expression levels of ADAMDEC1 were measured using an enzyme-linked immunosorbent assay kit (Cusabio, China), in accordance with the manufacturer’s instructions.

To investigate if the secretion of ADAMDEC1 into the synovial fluid was a biomarker to discriminate RA from OA, the AUC, and the sensitivity and specificity, were calculated as described above.

### Statistical analysis

The DEGs in the datasets investigated were analyzed using the limma, edgeR, and DESeq2 packages in R software. The significance of differences in ADAMDEC1 protein level in synovial fluid between RA and OA patients was determined via t-test using SPSS v26 (IBM, USA). *P*-values < 0.05 were considered statistically significant.

## Results

### Identification of DEGs between RA and OA in tissue from datasets

**Fig. 1:**
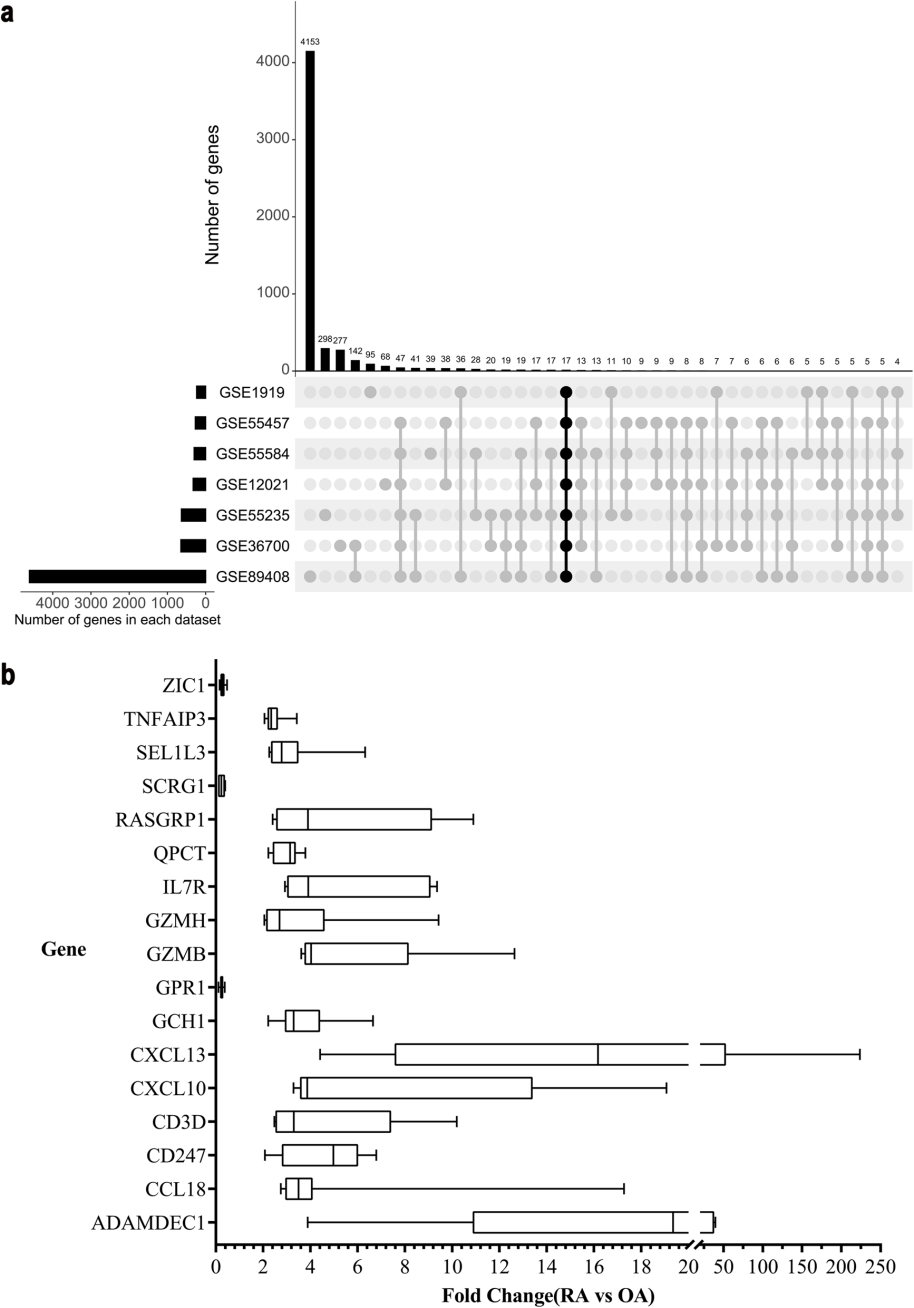
Identification of 17 DEGs between RA and OA. **a** Histogram indicating the number of overlapping DEGs expressed between RA and OA tissues. The bar chart above shows the DEGs between RA and OA in each type of intersection. The dotted line at the bottom right shows the types of events included in each type of intersection. 17 DEGs were identified in seven transcription profile datasets (GSE55235, GSE55457, GSE55584, GSE1919, GSE36700, GSE12021, and GSE89408) using package UpSetR. **b** A chart of 17 genes expressed differentially between RA and OA from all datasets (*P* < 0.05)

In the present study, a total of 311 samples of synovial tissue were obtained from datasets, of which 87 samples were from OA patients and 224 were from RA patients (supplementary table 1). Differential expression analysis between OA and RA synovial tissue samples was conducted using the seven datasets obtained from GEO (Fig. 1a). A total of 17 DEGs were identified that displayed significantly different expressions in RA compared with OA samples. In brief, 14 genes were upregulated and 3 were downregulated (Fig. 1b) in RA samples compared with OA samples.

### Discrimination power of DEGs based on the ROC curve

The discrimination power of the significantly expressed genes was determined based on the AUC, specificity, and sensitivity (Table 1). Every gene of the 17 DEGs could distinguish RA from OA, with an AUC higher than 0.8. Of these, seven DEGs (*CXCL13*, *GPR1*, *CCL18*, *ADAMDEC1*, *GZMB*, *IL-7R*, and *RASGRP1*) had an AUC higher than 0.90, with a specificity higher than 85%. In particular, ADAMDEC1 was the most powerful gene from the database for the discrimination of RA from OA (AUC: 0.999; sensitivity: 100%; specificity: 97.83%).

**Table 1:** Diagnostic specificity, sensitivity, and AUC values of 17 DEGs as determined by ROC analysis

Genes	Threshold ^a^	Specificity, %	Sensitivity, %	AUC	AUC 95% CI
*ADAMDEC1*	7.1	97.8	100.0	0.999	0.996–1
*RASGRP1*	6.5	97.8	91.2	0.987	0.972–1
*GZMB*	6.7	93.4	94.7	0.982	0.964–1
*CXCL13*	7.5	91.3	91.2	0.946	0.903–0.989
*GPR1*	6.7	97.8	75.4	0.939	0.898–0.980
*IL7R*	8.4	87.0	87.7	0.918	0.863–0.971
*CCL18*	10.8	87.0	86.0	0.903	0.845–0.961
*CD247*	7.7	84.8	87.7	0.895	0.830–0.961
*GCH1*	8.6	95.0	71.9	0.895	0.840–0.954
*QPCT*	7.2	91.3	84.2	0.895	0.831–0.959
*GZMH*	8.3	87.0	84.2	0.889	0.812–0.966
*ZIC1*	10.6	87.0	94.7	0.877	0.794–0.960
*CXCL10*	9.4	91.3	75.4	0.869	0.798–0.940
*SCRG1*	8.9	87.0	79.0	0.858	0.776–0.939
*CD3D*	9.2	91.3	75.4	0.857	0.782–0.933
*TNFAIP3*	9.3	89.1	86.0	0.832	0.743–0.920
*SEL1L3*	9.2	87.0	75.4	0.812	0.720–0.901

*AUC* area under the curve, *CI* confidence interval, *ADAMDEC1* ADAM Like Decysin 1, *CD247* T-Cell Surface Glycoprotein CD3 Zeta Chain, *CD3D* T‑cell surface glycoprotein CD3 δ chain, *CXCL10* C-X-C Motif Chemokine Ligand 10, *CXCL13* C-X-C Motif Chemokine Ligand 13, *GCH1* GTP Cyclohydrolase 1, *GPR1* G Protein-Coupled Receptor 1, *GZMB* Granzyme B, *GZMH* Granzyme B, *IL7R* interleukin 7 receptor, *QPCT* Glutaminyl-Peptide Cyclotransferase, *RASGRP1* RAS Guanyl Releasing Protein 1, *SCRG1* Stimulator Of Chondrogenesis 1, *SEL1L3* SEL1L Family Member 3, *TNFAIP3* TNF Alpha Induced Protein 3, *ZIC1* Zic Family Member 1

^a^fold changes in RA compared with OA tissue

### GO enrichment analysis of the DEGs

GO analysis demonstrated that the DEGs were principally enriched in BP, MF, and CC terms (Table 2). The BP terms involved in the 17 DEGs were primarily as follows: “T cell differentiation, cell mediated immunity” “inflammatory response” and “response to stimuli” The roles of the 17 DEGs in MF involved activation of “chemokine activity” promotion of “chemokine receptor binding” and regulation of “cytokine activity, receptor binding” and “G protein-coupled receptor binding” The CC terms of the 17 DEGs were related to components primarily located on the “external side of plasma membrane” “clathrin-coated vesicle membrane” and “T cell receptor complex”

**Table 2:** GO enrichment analysis of differentially expressed genes between rheumatoid arthritis and osteoarthritis

ID	GO term description	P	Genes
Biological processes (BPs)			
GO:0,060,326	chemotaxis, cell migration	0.015	*CCL18*/*CXCL10*/*CXCL13*
GO:0,001,906	cytotoxicity, cell killing	0.022	*GZMB*/*IL7R*/*RASGRP1*/*GZMH*
GO:0,002,544	inflammatory response	0.022	*CXCL13*/*TNFAIP3*/*CD247*/*CCL18*/*GCH1*
GO:0,071,216	response to stimulus	0.014	*CXCL10*/*CXCL13*/*GCH1*/*TNFAIP3*
GO:0,030,217	T cell differentiation, cell mediated immunity	0.023	*CD3D*/*IL7R*/*RASGRP1*/*CXCL13*/*TNFAIP3*/*GZMB*
Molecular functions (MFs)			
GO:0,008,009	chemokine activity	< 0.001	*CCL18*/*CXCL10*/*CXCL13*
GO:0,042,379	chemokine receptor binding	0.001	*CCL18*/*CXCL10*/*CXCL13*
GO:0,045,236	CXCR chemokine receptor binding	0.001	*CXCL10*/*CXCL13*
GO:0,048,020	CCR chemokine receptor binding	0.013	*CCL18*/*CXCL13*
GO:0,005,125	cytokine activity	0.014	*CCL18*/*CXCL10*/*CXCL13*
GO:0,001,664	G protein-coupled receptor binding	0.022	*CCL18*/*CXCL10*/*CXCL13*
GO:0,005,126	cytokine receptor binding	0.022	*CCL18*/*CXCL10*/*CXCL13*
Cellular component (CCs)			
GO:0,030,665	clathrin-coated vesicle membrane	0.036	*CD3D*/*IL7R*
GO:0,009,897	external side of plasma membrane	0.036	*CD3D*/*CXCL10*/*IL7R*
GO:0,042,101	T cell receptor complex	0.036	*CD247*/*CD3D*
GO:0,044,306	neuron projection terminus	0.036	*GCH1*/*SCRG1*
GO:0,030,662	coated vesicle membrane	0.043	*CD3D*/*IL7R*
GO:0,030,136	clathrin-coated vesicle	0.043	*CD3D*/*IL7R*

*GO* Gene Ontology, *ADAMDEC1* ADAM Like Decysin 1, *CD247* T-Cell Surface Glycoprotein CD3 Zeta Chain, *CD3D* T‑cell surface glycoprotein CD3 δ chain, *CXCL10* C-X-C Motif Chemokine Ligand 10, *CXCL13* C-X-C Motif Chemokine Ligand 13, *GCH1* GTP Cyclohydrolase 1, *GPR1* G Protein-Coupled Receptor 1, *GZMB* Granzyme B, *GZMH* Granzyme B, *IL7R* interleukin 7 receptor, *QPCT* Glutaminyl-Peptide Cyclotransferase, *RASGRP1* RAS Guanyl Releasing Protein 1, *SCRG1* Stimulator Of Chondrogenesis 1, *SEL1L3* SEL1L Family Member 3, *TNFAIP3* TNF Alpha Induced Protein 3, *ZIC1* Zic Family Member 1

### Determination of ADAMDEC1 expression in the synovial fluid

**Fig. 2:**
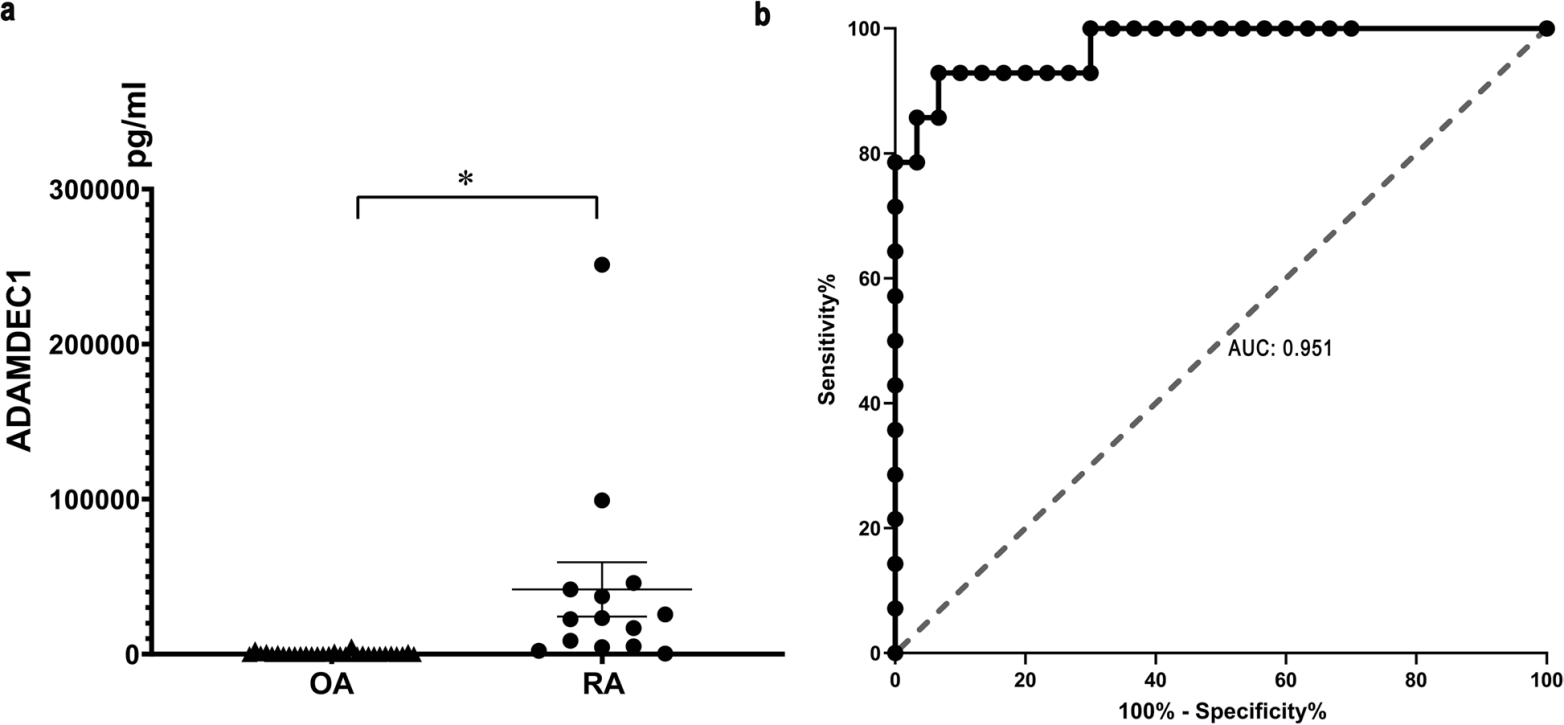
Expression of ADAMDEC1 in the synovial fluid from RA and OA patients. **a** ADAMDEC1 protein in RA and OA synovial fluid measured using enzyme-linked immunosorbent assay. **b** The ROC curve of ADAMDEC1 in the synovial fluid for distinguishing between RA and OA patients. *P < 0.05

Considering that ADAMDEC1 exhibited the greatest power of discrimination in the data analysis, it was predicted that the encoded protein would be expressed in its secretory form and, therefore, be detected in the synovial fluid. Thus, 44 synovial fluid samples from patients were collected in the present study, of which 14 samples were from patients with RA (RA group) and 30 were from patients with OA disease (OA group) (Table 3). Compared with the synovial fluid from the OA group, that from the RA group showed a significantly higher expression level of ADAMDEC (Fig. 2a). With the adoption of a cut-off value of 1957 pg/mL, ADAMDEC1 in the synovial fluid was able to discriminate RA patients from OA patients with an AUC value of 0.951, a specificity of 88.6%, and a sensitivity of 92.9% (Fig. 2b).

**Table 3:** Basic demographic characteristics of patients in validation

Features	Rheumatoid arthritis group	Osteoarthritis group
	(*n* = 14)	(*n* = 30)
Age (Mean ± SD)	66.3 ± 11.2	67.3 ± 7.3
Gender		
Female (%)	9 (64.3)	16 (53.3)
Male (%)	5 (35.7)	14 (46.7)
Disease duration, years (Mean ± SD)	11.4 ± 8.3	6.7 ± 4.7
Surgical site		
Articulatio Coxae (%)	6 (42.9)	5 (16.7)
Knee-joint (%)	8 (57.1)	25 (83.3)
Radiological imaging		
Degenerative lesions (%)	11 (78.6)	29 (96.7)
Joint effusion (%)	2 (14.3)	7 (23.3)

*SD* standard deviation

## Discussion

Although new potential therapeutic targets have been discovered with clinical significance in RA disease progression control, the treatment for OA and RA mainly relieves the symptoms when diseases progress into the late phase. The present study identified 17 DEGs between OA and RA samples in the database, among which one gene product was confirmed to be differently expressed between the RA and OA synovial fluid. The DEGs demonstrated considerable potential for the discrimination of RA from OA, and may be involved in disease pathogenesis; thus, this gene product may act as a new therapeutic target of RA in future.

Gene transcription and expression studies are now widely used to improve diagnosis and confirm novel pathways implicated in the pathogenesis of OA and RA [17, 18]. In previous work by other researchers, a number of DEGs in RA samples were identified in comparison with normal controls, based on limited datasets [19]. In addition, a number of DEGs have been identified in OA samples compared with normal controls [18–21]. Although it is not difficult for clinicians to distinguish patients with disease status (RA or OA) from healthy individuals, but those suffering from RA have symptoms similar to those with OA, and this results in difficulties in defining personalized treatment strategies for each disease group. While some work has been conducted to identify DEGs between OA tissues and RA tissues [6, 17, 22, 23], researchers have found large numbers of DEGs in small datasets in their studies. In the present study, a total of 17 DEGs were identified from seven datasets, reducing the number of candidate biomarkers for discriminating between OA and RA.

A number of DEGs in the present study, for instance, *CXCL13,* *CD247*, *GZMB*, *CCL18*, *IL7R*, and *ADAMDEC1* were identical to those reported by Li et al*.*, indicating their conservative role in RA pathogenesis [22]. However, no validation of clinical samples has been previously reported. In the present study, *ADAMDEC1* displayed the greatest discrimination between RA and OA in dataset analysis, and the transcription product was detected in the synovial fluid. In addition, the secretion of ADAMDEC1 in the synovial fluid also differed between RA and OA with good sensitivity and specificity. The sampling of synovial fluid is less invasive than that of synovial tissue for patients, indicating that ADAMDEC1 in the synovial fluid could represent a good biomarker for the discrimination of RA from OA.

In the present study, the role of ADAMDEC1 in identifying the presence of RA was indicated by dataset analysis of synovial tissue, eventually confirmed by protein expression in the synovial fluid. As a metalloprotease, ADAMDEC1 is not only associated with a number of inflammatory diseases (including pulmonary sarcoidosis, atherosclerosis, and Crohn’s disease) but it also plays a key role in the pathogenesis of cancer [24–26]. In brief, ADAMDEC1 promotes the proliferation, migration, and invasion of glioma, which is mediated by the downregulation of active caspase 3 and active caspase 9 [27]. In cancer stem cells, ADAMDEC1 solubilizes FGF2 to induce FGFR1, upregulating ADAMDEC1 expression to maintain its stemness [28]. Although the role of ADAMDEC1 has not been investigated in RA, its ability to induce inflammation in RA may be achieved by modulating the polarization of M1 macrophages as described in rosacea [29]. Evidence for the high expression of ADAMDEC1 in the synovial fluid has suggested its association with RA; thus, an investigation of the mechanism in the future might establish that it represents a potential target for RA treatment.

Although ADAMEDC1 was not involved in GO enrichment, other DEGs might play key roles in the regulation of RA pathogenesis. In the present study, several significantly expressed genes were found to be involved in immune response, chemokine‐mediated signaling pathway, and inflammatory response, indicating that RA is characterized by autoimmune and inflammatory processes similar to those studied previously [19]. Chemokines, cytokines, and their receptors play a fundamental role in the activation of monocytes and lymphocytes at the site of inflammation, especially in RA [23, 30]. A vicious cycle of changing chemokine levels and signal transduction pathways contributes to cartilage and bone destruction by RA synoviocytes [22, 31]. For example, CCL18 released from synovial macrophages and endothelial cells in RA synovial tissues may activate fibroblast-like synoviocytes and is partly responsible for the pathogenesis of RA [32].

There are also a number of limitations to the present study. First, the number of cases with which validation was conducted was small and from a single center, but the results actually confirm the differential expression of ADAMDEC1 in clinical samples. Second, the RA group studied here was actually derived from patients who received joint replacement, indicating that the progress of disease had not been efficiently controlled. In fact, our findings indicate that elevated ADAMDEC1 in the synovial fluid suggests the presence of RA lesions. The discriminative power for detecting RA lesion in early stage is necessary to be determined in future.

## Conclusions

In conclusion, bioinformatics analysis provided 17 potential markers in the synovial tissue for discriminating RA from OA with good sensitivity and specificity. After validation in clinical synovial fluid, ADAMDEC1 was found to be powerful enough to detect RA lesions even in patients who received joint replacement, indicating that it has a potential role as a biomarker for diagnosis and prognosis in the future.

## Supplementary Information

**Additional file 1: Supplemental Table 1.** Number of samples in downloadeddataset.

## Data Availability

All data generated or analyzed during the study are included in this published article and its supplementary information files.

## Abbreviations

RA: Rheumatoid arthritis
OA: Osteoarthritis
DEG: Differentially expressed genes
ROC: Receiver operating characteristic
AUC: Area under the ROC curve
GO: Gene Ontology
ADAMDEC1: ADAM Like Decysin 1
CD247: T-Cell Surface Glycoprotein CD3 Zeta Chain
CD3D: T‑cell surface glycoprotein CD3 δ chain
CXCL10: C-X-C Motif Chemokine Ligand 10
CXCL13: C-X-C Motif Chemokine Ligand 13
GCH1: GTP Cyclohydrolase 1
GPR1: G Protein-Coupled Receptor 1
GZMB: Granzyme B
GZMH: Granzyme H
IL7R: Interleukin 7 receptor
QPCT: Glutaminyl-Peptide Cyclotransferase
RASGRP1: RAS Guanyl Releasing Protein 1
SCRG1: Stimulator Of Chondrogenesis 1
SEL1L3: SEL1L Family Member 3
TNFAIP3: TNF Alpha Induced Protein 3
ZIC1: Zic Family Member 1
