# Supplementary material for: Potential biomarkers that discriminate rheumatoid arthritis and osteoarthritis based on the analysis and validation of datasets
Source: BMC Musculoskelet Disord. 2022 Apr 4;23:319. doi: 10.1186/s12891-022-05277-x (PMC8978354; doi:10.1186/s12891-022-05277-x)
Supplement: Supplementary file 1 — Additional file 1: Supplemental Table 1. Number of samples in downloadeddataset. [file 12891_2022_5277_MOESM1_ESM.docx]

Supplemental Table 1. Number of samples in downloaded dataset.

| Dataset | OA Synovial Tissue Samples | RA Synovial Tissue Samples |
| --- | --- | --- |
| GSE55235 | 10 | 10 |
| GSE55457 | 10 | 13 |
| GSE55584 | 6 | 10 |
| GSE12021 | 23 | 21 |
| GSE1919 | 5 | 5 |
| GSE36700 | 5 | 7 |
| GSE89408 | 22 | 152 |
